# Decisions in health technology assessment: should we speak with one voice?

**DOI:** 10.1186/s12961-018-0385-y

**Published:** 2018-11-15

**Authors:** Tracy Merlin

**Affiliations:** Adelaide Health Technology Assessment (AHTA), School of Public Health, University of Adelaide, Adelaide, South Australia 5005 Australia

**Keywords:** Health technology assessment, Regulation, Heterogeneity, Meta-analysis, Influenza

## Abstract

Decisions regarding the regulation of individual medicines differ from country to country. In the case of Relenza, Mulinari and Davis (*Health Res Policy Syst* 15:93, 2017) have suggested that these inconsistencies are primarily due to processes, statistical methodologies and technical capacity varying between regulatory agencies. They go on to name specific individuals involved in the evaluation of this anti-influenza medicine and imply that differences in the judgements of these individuals has affected public policy concerning the market access of this medicine.

This Commentary argues that what may appear as inconsistent decision-making may in fact be due to differences in the applicability of the evidence base to the local population and health system for which each regulator has responsibility. If health technology assessors are providing nuanced judgements on the effectiveness of a medicine for the local population, differences in regulation and reimbursement decisions are to be expected.

## Main text

### Making decisions that are right… for each jurisdiction

Health technologies encompass medicines, devices, medical tests, programmes and procedures – basically any form of health intervention. Whenever a new health technology is brought to market it invariably undergoes a process of assessment. Health technology assessment (HTA) occurs over the lifecycle of each new health technology. It is used to inform pre-market horizon scanning, regulatory decision-making, public funding decision-making (reimbursement), clinical practice guideline development, and disinvestment decision-making.

Within the regulatory domain, the 2017 article by Mulinari and Davis in *Health Research Policy and Systems* critiqued the apparent inconsistent decision-making between European and United States regulators regarding the marketing and labelling of an anti-influenza medicine, Relenza [1]. This inconsistent decision-making was attributed to different HTA practices undertaken by the United States Food and Drugs Administration (FDA) and the European Union (specifically, the Swedish Medical Products Agency (MPA) as the reference member state), particularly regarding differences in how the evaluation was conducted as well as differences in judgements as to whether a meta-analysis of trial data was appropriate or not.

Inconsistent decision-making is expected in HTA. In fact, it is common knowledge that HTA “*globalises the evidence and localises the decision*” [2]. Different decisions are entirely reasonable because the applicability of the evidence to the local health system and target population for the technology will vary. Mulinari and Davis [1] explain that the trial evidence submitted to support the listing of Relenza was heterogeneous. Three key trials were initially submitted – an American trial that showed negligible effects from the medicine, and European and southern hemisphere trials that demonstrated a small but significant treatment effect. There are many reasons why a treatment effect will vary from population to population. It can be due to the design and conduct of the trial, differences in the composition and characteristics of the trial populations (age, ethnicity, gender, comorbidities, etc.), and differences in clinical practice (e.g. co-administered therapies, hospital discharge practices). It is often the case that the treatment effects from the trials will all be in a similar direction, such as favourable to the medicine, but that the magnitude of the effect will vary. When the magnitude of the effect is small, in some trials, no clinical benefit will be observed; alternatively, there may be a treatment effect modifier in one or more trial populations that nullifies or reverses the observed treatment effect.

This heterogeneity, or difference in results between studies, is common, and makes the decision to pool the results of studies in a meta-analysis a matter of judgement. In fact, Ioannidis et al. [3] found that, although statistical and clinical heterogeneity were the main reasons why a pooled estimate was not produced in 135 systematic reviews that could have meta-analysed the data, there was very large disagreement over what actually constituted heterogeneity. People judge heterogeneity differently.

The FDA was appropriately cautious about allowing Relenza to be marketed given the lack of clinical benefit observed in the American trial population. The situation was reversed for the MPA as a clinical benefit was observed in the European trial population. Because of differences in the applicability of the trial evidence to each regulatory agency’s local circumstances, it would appear that they also held different views as to whether the findings from each of the trials should be meta-analysed. According to Mulinari and Davis [1], the FDA chose not to accept a meta-analysis of the trial results because of the observed heterogeneity between the trials and concerns about applicability of the pooled result to the American population, noting that Relenza had proved largely ineffective in American patients in whom relief medication was used more frequently. This assessment by the FDA is entirely reasonable.

Conversely, the Swedish MPA did accept a meta-analysis of the trial results and, in their context, this is also reasonable. The European trial found a median time reduction of 2.5 days to symptom improvement in influenza-positive patients receiving Relenza; therefore, by allowing the meta-analysis of the trials, the pooled result dropped to 1.5 days, which is a more conservative estimate of the treatment effect for the European population and in accordance with the variability in results observed among all the trials.

Each regulatory agency appears to have appropriately interpreted the evidence supplied to them in the context of their own population and health system.

### Responsibility for the decision

Particular individuals from both regulatory agencies were named in the article by Mulinari and Davis [1]. In Letters to the Editor in this issue of the journal, the MPA has objected to having their assessor named in the article [4], and Mulinari and Davis [5] have responded with a reaffirmation of their right to name these individuals because their identities are a matter of public record. The authors have argued that, as experts that influence public policy decisions, the identity of these individuals and their work should be open to public scrutiny [5].

However, often the work of HTA assessors is de-identified to protect them from the large, vested interests associated with the technologies they are assessing; otherwise, there would be a large power imbalance. Similar to government public servants, for HTA assessors, the body responsible for the technology assessment and subsequent decisions is the agency, not the individual. The HTA team evaluates the medicine on behalf of the agency. As decisions informed by that evaluation can have multi-million-dollar impacts, the agency handles any associated media and legal ramifications and is ultimately responsible for the quality and credibility of the evaluation.

What is clear from the Mulinari and Davis [1] analysis is that there are different levels of resourcing for regulatory assessment between the MPA and FDA, with many more staff (larger teams) with extensive specialist knowledge available for FDA reviews of medicines. Is it then appropriate for individuals to be singled out and publicly identified for activities that are undertaken and constrained by the employing agency’s own policies and resourcing?

The identity of the MPA assessor in the paper by Mulinari and Davis [1] was obtained through a Freedom of Information request; it was not freely available to the public. It is to the MPA’s credit that they have indicated that they, as an agency, are responsible for the assessment and decisions made regarding Relenza, and that these should not be attributed to a particular individual and that the individual should not have been named [4].

The large FDA assessment team responsible for the assessment of Relenza is a matter of public record but, being a large team, the accountability is shared. Although Mulinari and Davis [1] lament the lack of engagement in public discourse by regulators to communicate the results of their technology assessments, publicly naming individual assessors is unlikely to facilitate this.

### Conclusions

HTA assessments are performed by a team, with the HTA agency being ultimately responsible for the technology evaluation and ensuing recommendations. The analysis from Mulinari and Davis [1] would have lost none of its rigour and argument had they refrained from naming names. Differences in resourcing and evaluation capacity in regulatory agencies may affect how an evaluation is performed but does not necessarily equate to poor decision-making. Given that HTA ‘globalises the evidence and localises the decision’ it is not uncommon for there to be differences in decision-making across jurisdictions, populations and health systems. Both the MPA and FDA were justified in relying on the evidence that was most applicable to their marketing situation.

## Abbreviations

FDA: Food and Drugs Administration
HTA: Health Technology Assessment
MPA: Medical Products Agency
